# Assessment of Paclitaxel Drug-Coated Balloon-Only Angioplasty for Stent Thrombosis: SPARTAN-ST Study

**DOI:** 10.3390/jcdd12020059

**Published:** 2025-02-05

**Authors:** Ioannis Merinopoulos, Bhalraam U, Tharusha Gunawardena, Natasha Corballis, Rajkumar Natarajan, Upul Wickramarachchi, Clint Maart, Sulfi Sreekumar, Chris Sawh, Johannes Reinhold, Trevor Wistow, Alisdair Ryding, Timothy Gilbert, Vassilios S. Vassiliou, Simon C. Eccleshall

**Affiliations:** 1 Department of Cardiology, Norfolk and Norwich University Hospital, Colney Ln, Norwich NR4 7UY, UK; bhalraam.u@nnuh.nhs.uk (B.U.); tharusha.gunawardena@gmail.com (T.G.); natasha.corballis@nnuh.nhs.uk (N.C.); rajkumar.natarajan@nnuh.nhs.uk (R.N.); mruwii@gmail.com (U.W.); clint.maart@nnuh.nhs.uk (C.M.); sreekumar.sulfi@nnuh.nhs.uk (S.S.); chris.sawh@nnuh.nhs.uk (C.S.); johannes.reinhold@nnuh.nhs.uk (J.R.); trevor.wistow@nnuh.nhs.uk (T.W.); alisdair.ryding@nnuh.nhs.uk (A.R.); timothy.gilbert@nnuh.nhs.uk (T.G.); v.vassiliou@uea.ac.uk (V.S.V.); simon.eccleshall@nnuh.nhs.uk (S.C.E.); 2 Norwich Medical School, University of East Anglia, Norwich NR4 7TJ, UK

**Keywords:** DCB, stent thrombosis

## Abstract

Background: There are no data regarding the outcomes of patients with stent thrombosis (ST) being treated with drug-coated balloon (DCB) angioplasty. Our aim was to compare the outcomes of patients with ST treated with DCB vs. a drug eluting stent (DES). Methods: In this registry analysis, we identified all patients treated for ST in our institution from June 2011 until November 2019. We excluded patients who died in the cath lab, patients with uncrossable lesions, and patients treated with thrombectomy only. Patient outcomes were obtained from Hospital Episodes Statistics from NHS England. The primary endpoint of this study was the composite of cardiovascular mortality, acute coronary syndrome, or target lesion revascularisation. The data were analysed with Cox regression and Kaplan–Meier estimator plots. Results: A total of 173 patients were identified; 92 treated with DCB-only, 36 with balloon angioplasty (BA), 26 with DES, and 19 with a combination of DES and DCB. We compared the outcomes of 92 patients with DCB versus 20 patients with DES, all of which had presented with late or very late ST. There was no difference between DCB and DES in terms of the primary endpoint (*p* = 0.06). Multivariate analysis identified diabetes (adverse) and the use of GPIIbIIIa inhibitor (favourable) as the only independent predictors of the primary endpoint. Implantation of a DES was independently associated with worse cardiovascular mortality. Conclusions: This is the first study assessing the outcomes of patients with ST treated with DCB only. It has demonstrated that DCBs are an attractive therapeutic option with a tendency towards favourable outcomes when compared to DESs.

## 1. Introduction

Stent thrombosis (ST) is a rare but potentially catastrophic complication of coronary stent implantation. It is associated with significant mortality and morbidity; therefore, a substantial effort has been undertaken to understand its pathophysiology, reduce its incidence, and define its appropriate management. Despite great advances in pharmacotherapy and stent design and implantation techniques over the years, ST remains an important, long-term risk even in the era of new-generation drug eluting stents (DESs) [1,2,3,4,5]. Due to its low incidence, there is a lack of large randomised clinical trials aimed at identifying the best treatment strategy for ST. Currently, therefore, appropriate management steps and guidelines are based on registry and observational studies [3,6].

Drug-coated balloons (DCBs) are an established treatment option for patients with in-stent restenosis (ISR) with a growing amount of data to support use in de novo disease; but has not yet been evaluated in the treatment of ST [7,8,9,10,11,12]. The purpose of our study was to compare the outcomes of patients with ST treated with DCB-only angioplasty as compared to DESs.

## 2. Methods

**Figure 1:**
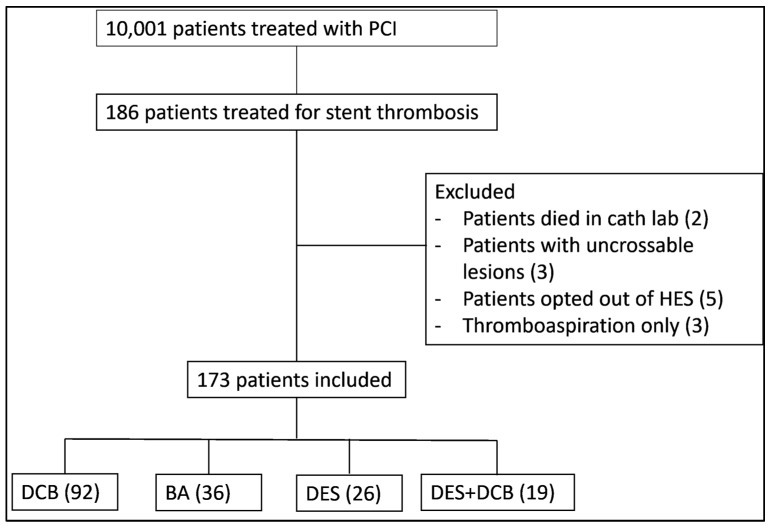
Study consort showing the flow of patients in this study. PCI: percutaneous coronary intervention, HES: hospital episode statistics, DCB: drug-coated balloon, BA: balloon angioplasty, DES: drug eluting stent.

The aSsessment of PAclitaxel dRug coaTed balloon only ANgioplasty for Stent Thrombosis (SPARTAN-ST) study was an investigator-initiated, single-centre, cohort study. In our institution, patients treated with percutaneous coronary intervention (PCI) are collated prospectively in a dedicated database. Following approval from the Northwest Haydock research ethics committee and institutional approval from the Norfolk & Norwich University Hospital and the University of East Anglia ethics committee, we retrospectively surveyed our prospective clinical database to identify all patients treated for ST between June 2011 and November 2019. The confidentiality advisory group waived the necessity for patient consent due to the retrospective nature of our study. The study protocol conforms to the ethical guidelines of the 1975 Declaration of Helsinki as reflected in a priori approval by our institution’s human research committee. We excluded patients who died in the catheterisation laboratory at the time of the index procedure, patients with uncrossable lesions and patients who were treated with thrombectomy only due to very low numbers (Figure 1). (Registration: https://clinicaltrials.gov/ct2/show/NCT04482972 Unique identifier: NCT04482972).

The primary endpoint was the composite endpoint of cardiovascular mortality, any acute coronary syndrome (ACS), or target lesion revascularisation (TLR) according to Academic Research Consortium definitions [13]. The secondary endpoints were all-cause mortality and the individual components of the primary endpoint. Patient outcomes were obtained from the Hospital Episodes Statistics from NHS digital, a data warehouse containing information of all admissions, outpatient, and accident and emergency attendances at all NHS hospitals in England. The ICD-10 diagnostic codes used to extract patients’ outcomes are provided in Supplementary Table S1. Classification of deaths as cardiovascular or non-cardiovascular was performed by two blinded adjudicators (IM, TG) according to the academic research consortium 2 consensus. The frailty index was calculated according to the validated Hospital Frailty Risk Score from the ICD-10 diagnostic codes [14]. Clinical and angiographic data were obtained from our prospectively collated database and supplemented with data from electronic records where required. All coronary angiograms were reviewed by two experienced operators (IM, TG) to confirm ST, thrombolysis in myocardial infarction (TIMI) flow pre- and post- intervention, and to identify bifurcation lesions. In case of disagreement, the angiogram was reviewed by a third operator before consensus was reached. The vessel diameter was considered as the largest pre/post-dilatation balloon or stent used, while lesion length was considered as the balloon, DCB, or stent length. The angiograms of all patients with repeat PCI were reviewed to identify unplanned TLR. Based on the timing of stent thrombosis, late ST is defined as 30 days < ST < 1 year from index stent, while very late ST is defined as ST > 1 year from index stent implantation [15].

### Statistical Analysis

All statistical analyses were performed using R version 4.1.0 (R Foundation for Statistical Computing, Vienna, Austria). Baseline characteristics were summarised using descriptive statistics. Continuous variables were presented as medians and interquartile ranges (IQRs), while categorical variables were presented as counts and percentages. Comparisons between groups were made using the Wilcoxon rank-sum test for continuous variables and Fisher’s exact test or Pearson’s Chi-squared test for categorical variables, as appropriate.

Survival analyses were conducted using Kaplan–Meier estimator plots and log-rank tests to compare outcomes between DCB and DES groups. Cox proportional hazard models were used to estimate hazard ratios (HRs) and 95% confidence intervals (CIs) for both univariate and multivariate analyses. The multivariate models were adjusted for potential confounders including age, gender, glomerular filtration rate (GFR), history of cerebrovascular events, myocardial infarction, smoking status, frailty group, presentation (STEMI vs. NSTEMI), diabetes mellitus, peripheral vascular disease, mechanical ventilation, and use of glycoprotein IIb/IIIa inhibitors.

For the primary composite endpoint of cardiovascular mortality, ACS or TLR, as well as for individual components and other secondary endpoints, we performed both univariate and multivariate Cox regression analyses. The proportional hazards assumption was verified using Schoenfeld residuals.

To account for potential confounding, we used a standardised set of covariates across all multivariate models. These included treatment strategy (DCB vs. DES), demographic factors (age, gender), clinical factors (GFR, history of cerebrovascular events, myocardial infarction, smoking status, frailty, presentation, diabetes, peripheral vascular disease), and procedural factors (mechanical ventilation, use of glycoprotein IIb/IIIa inhibitors). We selected covariates for regression based on three criteria: (1) variables demonstrating statistical significance (*p* < 0.10) in univariate analysis (diabetes, GP IIb/IIIa inhibitor use), (2) variables showing significant baseline differences between groups (CABG history, GFR, vessel diameter), and (3) clinically relevant variables from previous stent thrombosis studies, regardless of statistical significance (age, gender, cardiovascular risk factors). Given our sample size, we limited the total number of covariates to avoid overfitting while maintaining model stability.

All statistical tests were two-tailed, and a *p*-value < 0.05 was considered statistically significant. To address multiple comparisons, we applied the Benjamani–Hochberg procedure to control the false discovery rate.

The analysis scripts and output were generated using the tidyverse, survival, survminer, and gtsummary packages in R. Kaplan–Meier plots were created using the ggsurvplot function from the survminer package, with risk tables included to show the number of patients at risk at different time points.

## 3. Results

**Figure 2:**
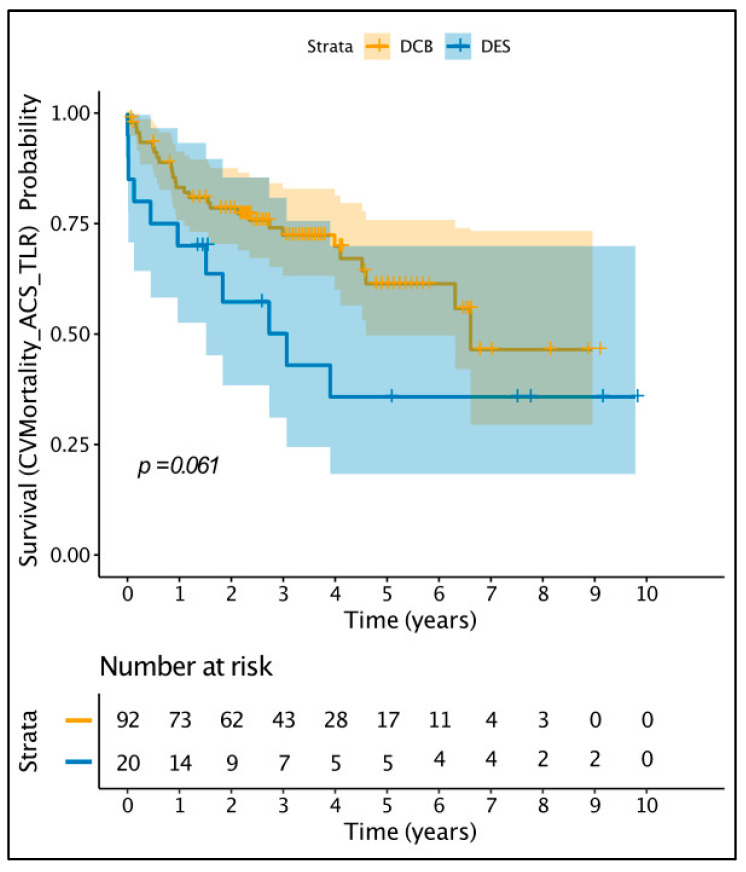
Kaplan–Meier estimator plot for the primary composite endpoint.

A total of 173 patients were identified; 92 were treated with DCB, 36 with balloon angioplasty (BA), 26 with DES, and 19 with a combination of DES and DCB. DCB-only angioplasty necessitates optimal vessel preparation prior to final treatment of the vessel with DCB, in a standard fashion as described in the DCB consensus document [10]. The patient baseline characteristics and clinical and angiographic characteristics of the full cohort are provided in Supplementary Table S2, while Supplementary Tables S3 and S4 provide the characteristics of patients with late or very-late stent thrombosis. As all patients treated with DCB-only angioplasty had late or very-late stent thrombosis, we elected to continue data analysis including only patients with late or very-late stent thrombosis, comparing DCB-only vs. DES, which were the two largest groups. The DCB used was paclitaxel-based (SeQuent Please or SeQuent Please NEO) while the great majority of DES used was second generation (85%). The use of DCB-only angioplasty, including ISR, stent thrombosis, de novo disease steadily increased over the time period of our study, as our operators became more experienced and comfortable with this technology. Table 1 demonstrates the baseline patient characteristics, while Table 2 demonstrates the clinical and angiographic characteristics. The median age was 67 (57–74) years old and 79% of patients were male. There were very few differences in the baseline patient characteristics. The DES group had significantly more patients with a history of coronary artery bypass and significantly lower estimated glomerular filtration rate. However, the median eGFR for both groups were in the mildly decreased range (>60 mL/min/1.73 m^2^). In terms of the clinical and angiographic characteristics, the DCB group had significantly more patients with very late stent thrombosis, while the DES group had larger vessel diameters. However, the vessel diameters of both groups were in the large-vessel category. After a median follow up of 3.5 years, the primary combined endpoint of cardiovascular mortality or ACS or TLR occurred in 29 patients (32%) in the DCB-only group vs. 11 patients (55%) in the DES group (*p* = 0.06), as shown in the Kaplan–Meier estimator plot (Figure 2). Univariate Cox regression analysis identified diabetes and intubation as adverse prognostic indicators of the primary endpoint while use of GPIIb/IIIa inhibitors and presentation with very late stent thrombosis were associated with better prognosis (Supplementary Table S5).

Multivariate Cox regression analysis demonstrated that diabetes was the only independent poor prognostic indicator, while use of GP IIb/IIIa inhibitor was the only independent good prognostic indicator (Table 3).

In terms of the secondary endpoints, multivariate Cox regression analysis identified (a) frailty as the only independent poor prognostic indicator of all-cause mortality, (b) DES implantation and peripheral vascular disease as the only independent poor prognostic indicators of cardiovascular mortality, and (c) the use of GP IIb/IIIa inhibitor as the only independent good prognostic indicator for ACS or TLR (Table 4).

**Figure 3:**
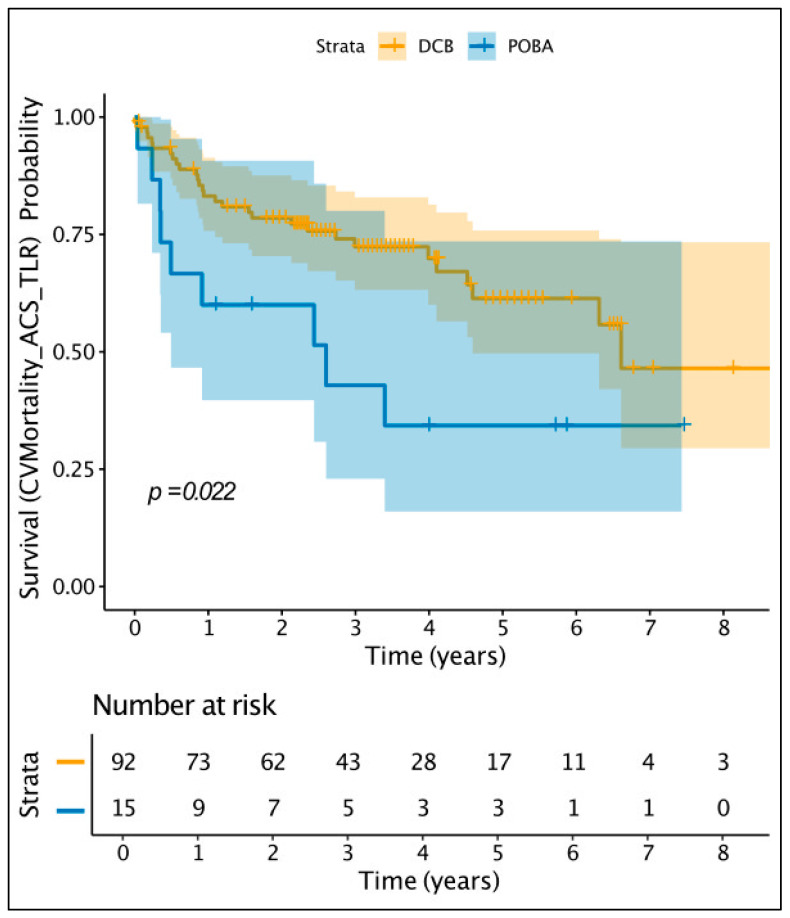
Kaplan–Meier estimator plot for DCB vs. POBA for primary endpoint.

Furthermore, we compared DCB vs. POBA in patients with late or very-late ST, aiming to explore if there is a benefit of DCB in addition to POBA. As demonstrated in Supplementary Table S6, the groups were very well balanced in terms of clinical and angiographic characteristics. The only difference was the lesion length, which was significantly longer in the DCB group (26 vs. 20 mm, *p* = 0.002). Kaplan–Meier estimator plot analysis (Figure 3) demonstrated a significant difference between DCB and POBA in favour of DCB, in terms of the combined endpoint of cardiovascular mortality or ACS or TLR (*p* = 0.022), driven mainly by the TLR.

## 4. Discussion

Stent thrombosis, with a 5–45% mortality and 15–20% recurrence rate at 5 years, represents the most severe end of the stent failure spectrum [16]. It is a rare complication, but given the very large number of stent implantations worldwide annually, is responsible for significant mortality and morbidity [16]. Over time, our understanding of the pathophysiology and risk factors for ST has increased significantly and led to improved pharmacotherapy, stent design, and implantation techniques [17,18,19]. However, the rarity of ST has limited the design of studies evaluating specific therapies, whilst prevention appears to be the only effective treatment [5]. This is the first study comparing DCB-only versus DES for patients with late or very late stent thrombosis. Our study suggests that use of GP IIb/IIIa inhibitor is the only independent good prognostic indicator, while history of diabetes is the only independent poor prognostic indicator of the composite endpoint of cardiovascular mortality, ACS or TLR. Importantly, our study also identified treatment strategy with DES implantation as an independent poor prognostic factor for cardiovascular mortality. Furthermore, our study has also demonstrated a significant difference in favour of DCB compared to POBA for the primary outcome, driven mainly by TLR.

The results of our study are consistent with the ESTROFA registry, which had demonstrated that (a) the use of abciximab was independently associated with a reduction in ST recurrence and that (b) the implantation of a new stent was independently associated with worse mortality and recurrent ST [20]. Use of GP IIb/IIIa inhibitors, as well as thrombectomy, is encouraged by recent consensus guidelines, as ST is usually associated with high thrombotic burden and distal embolization [6]. Previous studies have also reported that the implantation of a new stent is associated with worse clinical outcomes, leading to a recommendation against systematic repeat stenting, especially in the setting of multiple stent layers [21,22].

There is a lack of randomised control trials evaluating the management of patients presenting with ST. Liberal use of intracoronary imaging is encouraged to identify pathophysiological factors leading to ST or stent-related factors that could be optimised with BA or further stent implantation. However, routine repeat stenting is discouraged [3,6]. The results of our study support the use of DCB over BA over routine stenting. Very late ST is a complex, multifactorial pathophysiological entity, only partially understood. Intravascular ultrasound and histopathological analysis of thrombus aspirates have shown that chronic inflammation and hypersensitivity reactions relate to incomplete stent apposition and formulate the pathophysiological substrate for very late ST [23]. ISR with superimposed thrombus has also been observed as a pathological mechanism leading to very late ST [23]. Two recent optical coherence tomography studies have demonstrated (a) that neoatherosclerosis is the responsible pathological substrate in about 25–30% of very late ST cases, (b) that there are similar mechanisms in very late ST in early- and new-generation DES, and (c) that there is an association between ST and ISR [24,25].

DCB is an evolving PCI option with class IA recommendation for the treatment of ISR [26]. It allows for the homogeneous transfer of antiproliferative drugs into the vessel wall via a lipophilic matrix without leaving a permanent implant behind. A somewhat lower efficacy compared with DES in the treatment of DES-ISR might be related to the complex underlying tissue substrate (neointimal hyperplasia in combination with neoatherosclerosis in DES-ISR), but is confounded by lesion preparation techniques and angiographic end point selection [10,27]. Even so, DCB angioplasty with liberal use of intracoronary imaging to guide meticulous lesion preparation, instead of repeat stenting, remains an attractive PCI option especially in patients with multiple previous stent layers [10]. The value of DCB-only angioplasty has also been investigated in patients with ST elevation myocardial infarction, which, similarly to stent thrombosis, is usually characterised by high thrombus burden [7,28]. Studies have shown that DCB-only angioplasty is a viable treatment option for patients with STEMI, avoiding concerns such as vessel sizing in the setting of vasoconstriction or high clot burden and unclear antiplatelet adherence [7,28].

To date, there have been only case reports describing the use of DCB for the treatment of very late ST [29]. Our study is the first to report the outcomes of patients with stent thrombosis and compare them with DES. It has demonstrated that in the context of late or very-late ST, DCB might be an attractive therapeutic option with favourable long-term clinical outcomes compared to further DES implantation, avoiding multiple stent layers.

## 5. Limitations

The retrospective, non-randomised nature of this work from a single centre is a potential source of bias. However, our institution is a large tertiary centre providing cardiac services to a population in excess of one million, with one of the highest implantation rates of DCB for coronary artery disease in the UK, and we incorporated all consecutive patients fulfilling the inclusion criteria [30]. Even though our study is retrospective and non-randomised, our clinical database was completed prospectively. The limited sample size, due to the rarity of stent thrombosis, is a limitation of our study, but we included all patients meeting our inclusion criteria. A specific effort was made to mitigate the differences between the groups by comparing DCB-only versus DES in patients with late or very-late stent thrombosis, which represent the largest groups. Furthermore, the lack of QCA assessment or data on left ventricular function represent limitations of our study.

## 6. Conclusions

In conclusion, this is the first study to compare patient outcomes of ST treated with DCB-only versus DES. It shows a tendency towards favourable long-term outcomes of patients with DCB-only angioplasty for late or very-late ST.

## Figures and Tables

**Table 1:** Baseline patient characteristics.

Characteristic	Overall, *n* = 112	DCB Only, *n* = 92	Stent, *n* = 20	*p*-Value ^1^
Gender, n (%)				0.37
Male	88 (79)	74 (80)	14 (70)	
Age, median (IQR)	67 (57–74)	66 (55–73)	69 (62–76)	0.30
Hypercholesterolaemia n (%)	65 (58)	51 (55)	14 (70)	0.23
HTN, n (%)	65 (58)	50 (54)	15 (75)	0.090
PVD, n (%)	13 (12)	12 (13)	1 (5.0)	0.46
Stroke n (%)	11 (9.8)	10 (11)	1 (5.0)	0.69
MI, n (%)	89 (79)	75 (82)	14 (70)	0.36
CABG, n (%)	8 (7.1)	4 (4.3)	4 (20)	**0.033**
Heart failure, n (%)	3 (2.7)	3 (3.3)	0 (0)	>0.99
AF, n (%)	13 (12)	11 (12)	2 (10)	>0.99
FHx of CAD, n (%)	18 (16)	14 (15)	4 (20)	0.74
COPD, n (%)	8 (7.1)	7 (7.6)	1 (5.0)	>0.99
Diabetes, n (%)	37 (33)	30 (33)	7 (35)	0.84
Smoking history, n (%)				0.29
Never smoked	18 (16)	13 (14)	5 (21)	
Current/Ex smoker	94 (84)	79 (88)	15 (79)	
GFR, median (IQR)	83 (63–99)	87 (68–100)	66 (52–81)	**0.013**
Frailty score, median (IQR)	0.00 (0.00–1.18)	0.00 (0.00–0.63)	0.00 (0.00–1.45)	0.18

HTN: hypertension, PVD: peripheral vascular disease, MI: myocardial infarction, CABG: coronary artery bypass graft, AF: atrial fibrillation, CAD: coronary artery disease, COPD: chronic obstructive pulmonary disease, GFR: glomerular filtration rate. ^1^ Wilcoxon rank sum test; Fisher’s exact test; Pearson’s Chi-squared test; Wilcoxon rank sum exact test.

**Table 2:** Clinical and angiographic characteristics.

Characteristic	Overall, *n* = 112	DCB Only, *n* = 92	Stent, *n* = 20	*p*-Value ^1^
Presentation, n (%)				0.27
STEMI	98 (87.5)	82 (89)	16 (80)	
NSTEMI	14 (12.5)	10 (11)	4 (20)	
Antiplatelet adherence, n (%)				
Nil reported	105 (93.7)	86 (93.5)	19 (95)	
Issues reported	7 (6.3)	6 (6.5)	1 (5.0)	
Timing for stent thrombosis, n (%)				**0.033**
Late	8 (7.1)	4 (4.3)	4 (20)	
Very late	104 (92.9)	88 (95.7)	16 (80)	
Cardiogenic shock, n (%)	12 (11)	8 (8.7)	4 (20)	0.22
Intubation, n (%)	5 (4.5)	3 (3.3)	2 (10)	0.22
Cardiac arrest, n (%)	16 (14)	13 (14)	3 (15)	>0.99
Vessel treated, n (%)				0.12
LAD	48 (42.8)	40 (43.5)	8 (40)	
LCx	21 (18.8)	17 (18.5)	4 (20)	
RCA	38 (33.9)	33 (35.8)	5 (25)	
Graft	5 (4.5)	2 (2.2)	3 (15)	
True bifurcation, n (%)	47 (42)	39 (42)	8 (40)	0.84
**Procedural characteristics**				
Gp IIbIIIa, n (%)	84 (75)	68 (74)	16 (80)	0.78
Thromboaspiration, n (%)	62 (55)	50 (54)	12 (60)	0.64
Intravascular imaging, n (%)				0.091
OCT	33 (29)	31 (34)	2 (10)	
IVUS	27 (24)	20 (22)	7 (35)	
Previous stent, n (%)				0.30
DES	56 (50)	47 (51)	9 (45)	
BMS	10 (8.9)	6 (6.5)	4 (20)	
DES and BMS	2 (1.8)	2 (2.2)	0 (0)	
BVS	0 (0)	0 (0)	0 (0)	
Unknown	44 (39.3)	37 (40.3)	7 (35)	
Vessel diameter, median (IQR)	3.50 (3.00–4.00)	3.50 (3.00–4.00)	4.00 (3.44–4.00)	**0.007**
Lesion length, median (IQR)	26 (20–39)	26 (20–38)	25 (19–40)	0.36
Heavy calcification	12 (11)	10 (11)	2 (10)	>0.99
Diffuse disease	19 (17)	16 (17)	3 (16)	>0.99
Tortuosity	12 (11)	11 (12)	1 (5.6)	0.69
TIMI flow pre, n (%)				0.84
0	80 (71.4)	66 (71.7)	14 (70)	
1	5 (4.5)	4 (4.3)	1 (5.0)	
2	12 (10.7)	9 (9.8)	3 (15)	
3	15 (13.4)	13 (14.2)	2 (10)	
TIMI flow post, n (%)				0.28
0	0 (0)	0 (0)	0 (0)	
1	1 (0.9)	1 (1.1)	0 (0)	
2	12 (10.7)	12 (13)	0 (0)	
3	99 (88.4)	79 (85.9)	20 (100)	

Table 2: Baseline clinical characteristics of patients treated for stent thrombosis. Data are n (%) and bold denotes significant results. Abbreviations: STEMI: ST elevation myocardial infarction, NSTEMI: non ST elevation myocardial infarction, LVSD: left ventricular systolic dysfunction, LAD: left anterior descending, LCx: circumflex, RCA: right coronary artery, GpIIbIIIa: glycoprotein IIb/IIIa, OCT: optical coherence tomography, IVUS: intravascular imaging, DES: drug eluting stent, BMS: bare metal stent, BVS: biovascular scaffold, TIMI: thrombolysis in myocardial infarction. ^1^ Wilcoxon rank sum test; Fisher’s exact test; Pearson’s Chi-squared test; Wilcoxon rank sum exact test.

**Table 3:** Multivariate Cox regression model for primary composite endpoint.

Cardiovascular Mortality/ACS/TLR (Multivariate)	HR (95% CI) ^1^	*p*-Value
DCB-only	0.49 (0.15 to 1.59)	0.24
Age	1.00 (0.97 to 1.04)	0.86
Male	0.79 (0.31 to 2.01)	0.63
GFR	1.01 (0.99 to 1.02)	0.46
Stroke	1.66 (0.47 to 5.81)	0.43
MI	2.90 (0.95 to 8.87)	0.061
Smoking history	0.76 (0.26 to 2.25)	0.62
Frailty group		
Low	—	
Intermediate	1.21 (0.21 to 7.03)	0.83
High	0.00 (0.00 to Inf)	>0.99
NSTEMI vs. STEMI (NSTEMI)	0.95 (0.29 to 3.11)	0.93
Diabetes	3.02 (1.40 to 6.50)	**0.005**
Peripheral vascular disease	1.64 (0.58 to 4.63)	0.35
Intubation	5.36 (0.91 to 31.7)	0.064
GP IIb/IIIa	0.42 (0.18 to 0.97)	**0.042**

^1^ HR = Hazard ratio, CI = Confidence interval.

**Table 4:** Independent predictors of primary and secondary outcomes following multivariate Cox regression analysis.

Outcome	Independent Predictors	HR (95% CI)	*p*-Value
Primary outcome (CV mortality/ACS/TLR)	Diabetes GP IIb/IIIa inhibitor	3.02 (1.40–6.50) 0.42 (0.18–0.97)	0.005 0.042
All-cause mortality	High frailty	146 (3.31–6410)	0.01
Cardiovascular mortality	DES implantation Peripheral vascular disease	1.02 (1.08–95.8) 40.2 (3.03–535)	0.043 0.005
Acute coronary syndrome	GPIIb/IIIa inhibitor	0.36 (0.13–1.00)	0.05
Target lesion revascularisation	GPIIb/IIIa inhibitor	0.24 (0.08–0.78)	0.017

## Data Availability

The data are available following appropriate request to the authors.

## Supplementary Materials

The following supporting information can be downloaded at https://www.mdpi.com/article/10.3390/jcdd12020059/s1, Supplementary Table S1: ICD-10 codes used to identify patients’ outcomes; Supplementary Table S2: Baseline patient characteristics for the full cohort; Supplementary Table S3: Baseline patient characteristics; Supplementary Table S4: Clinical and angiographic characteristics (Full cohort); Supplementary Table S5: Univariate Cox regression analysis for primary composite endpointl; Supplementary Table S6: Clinical and angiographic characteristics (DCB-only vs. POBA).
