# Supplementary material for: Assessment of Paclitaxel Drug-Coated Balloon-Only Angioplasty for Stent Thrombosis: SPARTAN-ST Study
Source: J Cardiovasc Dev Dis. 2025 Feb 5;12(2):59. doi: 10.3390/jcdd12020059 (PMC11856968; doi:10.3390/jcdd12020059)
Supplement: Supplementary file 1 [file jcdd-12-00059-s001.zip › jcdd-3365793-supplementary.pdf]

Supplementary Table S1: ICD-10 codes used to identify patients' outcomes

**Acute coronary syndrome**	
STEMI	I210* I211* I212* I213
NSTEACS	I214 I219 I200 (UA)
Re-infarction	I22*
**Stroke**	
Ischaemic stroke	I63*
Transient ischaemic attack	G459, G453
**Major bleeding**	
Subarachnoid haemorrhage	I60*
Intracerebral haemorrhage	I61*
Non-traumatic intracranial bleeding	I62*
Gastrointestinal bleeding	K920, K921, K922
Haemorrhage not elsewhere specified	R58X

Supplementary Table S1: ICD-10 codes used to identify patients' outcomes.

Supplementary Table S2: Baseline patient characteristics for the full cohort

**Characteristic**	**Overall**, N = 173	**DCB Only**, N = 92	**Stent**, N = 26	**POBA**, N = 36	**Stent and DCB**, N = 19	**p-value**[1]
Gender, n (%)						0.41
Female	41 (24)	18 (20)	6 (23)	12 (33)	5 (26)	
Male	132 (76)	74 (80)	20 (77)	24 (67)	14 (74)	
Age, Median (IQR)	68 (58 – 74)	66 (55 – 73)	69 (63 – 77)	70 (65 – 74)	63 (53 – 71)	0.087
Hypercholesterolaemia, n (%)	93 (54)	51 (55)	16 (62)	19 (53)	7 (37)	0.40
HTN, n (%)	94 (54)	50 (54)	20 (77)	15 (42)	9 (47)	**0.045**
Peripheral vascular disease, n (%)	15 (8.7)	12 (13)	2 (7.7)	1 (2.8)	0 (0)	0.16
Stroke, n (%)	15 (8.7)	10 (11)	2 (7.7)	3 (8.3)	0 (0)	0.61
Myocardial infarction, n (%)	136 (79)	75 (82)	20 (77)	28 (78)	13 (68)	0.59
CABG, n (%)	10 (5.8)	4 (4.3)	5 (19)	1 (2.8)	0 (0)	**0.029**
Heart failure, n (%)	5 (2.9)	3 (3.3)	0 (0)	2 (5.6)	0 (0)	0.75
Atrial fibrillation, n (%)	22 (13)	11 (12)	4 (15)	4 (11)	3 (16)	0.89
Family history of IHD, n (%)	33 (19)	14 (15)	4 (15)	9 (25)	6 (32)	0.27
COPD, n (%)	13 (7.5)	7 (7.6)	1 (3.8)	3 (8.3)	2 (11)	0.87
Diabetes, n (%)	49 (28)	30 (33)	8 (31)	7 (19)	4 (21)	0.42

**Characteristic**	**Overall**, N = 173	**DCB Only**, N = 92	**Stent**, N = 26	**POBA**, N = 36	**Stent and DCB**, N = 19	**p-value**[1]
Smoking status, n (%)						0.065
Never Smoked	40 (19)	13 (12)	8 (28)	13 (30)	5 (18)	
Current/Ex Smoker	134 (81)	79 (88)	18 (72)	23 (70)	14 (82)	
GFR, Median (IQR)	82 (68 – 99)	87 (68 – 100)	70 (52 – 81)	79 (68 – 93)	95 (76 – 114)	**0.005**
Frailty score, Median (IQR)	0.00 (0.00 – 0.80)	0.00 (0.00 – 0.63)	0.00 (0.00 – 1.40)	0.00 (0.00 – 0.50)	0.00 (0.00 – 0.65)	0.66
Presentation, n (%)						0.61
STEMI	152 (88)	82 (89)	22 (85)	30 (83)	18 (95)	
NSTEMI	21 (12)	10 (11)	4 (15)	6 (17)	1 (5.3)	
Timing for stent thrombosis, n (%)						
Acute	12 (6.9)	0 (0)	4 (15)	8 (22)	0 (0)	
Subacute	15 (8.7)	0 (0)	2 (7.7)	13 (36)	0 (0)	
Late	15 (8.7)	4 (4.3)	4 (15)	3 (8.3)	4 (21)	
Very Late	131 (76)	88 (96)	16 (62)	12 (33)	15 (79)	
Cardiogenic Shock, n (%)	19 (11)	8 (8.7)	6 (23)	2 (5.6)	3 (16)	0.11
Mechanical ventilation, n (%)	8 (4.6)	3 (3.3)	3 (12)	0 (0)	2 (11)	0.061

**Characteristic**	**Overall**, N = 173	**DCB Only**, N = 92	**Stent**, N = 26	**POBA**, N = 36	**Stent and DCB**, N = 19	**p-value**[1]
Cardiac Arrest, n (%)	22 (13)	13 (14)	5 (19)	3 (8.3)	1 (5.3)	0.48
Vessel treated, n (%)						
LMS	1 (0.6)	0 (0)	0 (0)	1 (2.8)	0 (0)	
LAD	82 (48)	40 (43)	10 (38)	24 (67)	8 (44)	
LCx	31 (18)	17 (18)	6 (23)	4 (11)	4 (22)	
RCA	53 (31)	33 (36)	7 (27)	7 (19)	6 (33)	
Graft	5 (2.9)	2 (2.2)	3 (12)	0 (0)	0 (0)	
Unknown	1	0	0	0	1	
True_Bif, n (%)	81 (47)	39 (42)	11 (42)	20 (56)	11 (58)	0.40
GpIIbIIIa, n (%)	132 (76)	68 (74)	22 (85)	27 (75)	15 (79)	0.75
Thromboaspiration, n (%)	102 (59)	50 (54)	17 (65)	22 (61)	13 (68)	0.57
Intravascular imaging, n (%)						
None	71 (41)	41 (45)	13 (50)	9 (25)	8 (42)	
OCT	54 (31)	31 (34)	4 (15)	15 (42)	4 (21)	
IVUS	45 (26)	20 (22)	9 (35)	9 (25)	7 (37)	
Both	3 (1.7)	0 (0)	0 (0)	3 (8.3)	0 (0)	
Previous stent, n (%)						0.086

**Characteristic**	**Overall**, N = 173	**DCB Only**, N = 92	**Stent**, N = 26	**POBA**, N = 36	**Stent and DCB**, N = 19	**p-value**[1]
DES	95 (55)	47 (51)	15 (58)	25 (69)	8 (42)	
BMS	15 (8.7)	6 (6.5)	4 (15)	3 (8.3)	2 (11)	
BVS	2 (1.2)	0 (0)	0 (0)	2 (5.6)	0 (0)	
unknown	59 (34)	37 (40)	7 (27)	6 (17)	9 (47)	
DES and BMS	2 (1.2)	2 (2.2)	0 (0)	0 (0)	0 (0)	
Vessel diameter, Median (IQR)	3.50 (3.00 – 4.00)	3.50 (3.00 – 4.00)	4.00 (3.50 – 4.00)	3.50 (3.00 – 3.75)	3.75 (3.25 – 4.00)	**0.004**
Lesion length, Median (IQR)	26 (20 – 38)	26 (20 – 38)	25 (20 – 38)	20 (15 – 30)	40 (32 – 58)	**<0.001**
TIMI flow pre, n (%)						0.96
0	125 (72)	66 (72)	19 (73)	26 (72)	14 (74)	
1	8 (4.6)	4 (4.3)	1 (3.8)	2 (5.6)	1 (5.3)	
2	19 (11)	9 (9.8)	4 (15)	3 (8.3)	3 (16)	
3	21 (12)	13 (14)	2 (7.7)	5 (14)	1 (5.3)	
TIMI flow post, n (%)						0.87
0	0 (0)	0 (0)	0 (0)	0 (0)	0 (0)	
1	2 (1.2)	1 (1.1)	0 (0)	1 (2.8)	0 (0)	
2	19 (11)	12 (13)	3 (12)	3 (8.3)	1 (5.3)	
3	152 (88)	79 (86)	23 (88)	32 (89)	18 (95)	

**Characteristic**	**Overall**, N = 173	**DCB Only**, N = 92	**Stent**, N = 26	**POBA**, N = 36	**Stent and DCB**, N = 19	**p-value**[1]
Aspirin, n (%)	168 (97)	90 (98)	25 (96)	35 (97)	18 (95)	0.69
P2Y12, n (%)						
Clopidogrel	48 (28)	21 (23)	14 (54)	7 (19)	6 (32)	
Ticagrelor	113 (65)	69 (75)	9 (35)	25 (69)	10 (53)	
Prasugrel	8 (4.6)	1 (1.1)	2 (7.7)	3 (8.3)	2 (11)	
No	4 (2.3)	1 (1.1)	1 (3.8)	1 (2.8)	1 (5.3)	
Anticoagulation, n (%)	13 (7.5)	9 (9.8)	0 (0)	4 (11)	0 (0)	0.20
Antiplatelet adherence, n (%)						0.67
No reported issues	156 (94)	81 (93.1)	25 (96.2)	34 (97.1)	16 (89)	
Issues Reported	10 (6.0)	6 (6.9)	1 (3.8)	1 (2.9)	2 (11)	

Supplementary Table S3: Baseline patient characteristics

**Characteristic**	**Overall**, N = 146	**DCB Only**, N = 92	**Stent**, N = 20	**BA**, N = 15	**Stent and DCB**, N = 19	**p-value**[1]
Gender, n (%)						0.61
Female	33 (23)	18 (20)	6 (30)	4 (27)	5 (26)	
Male	113 (77)	74 (80)	14 (70)	11 (73)	14 (74)	
Age, Median (IQR)	68 (56 – 74)	66 (55 – 73)	69 (62 – 76)	71 (68 – 74)	63 (53 – 71)	0.08
Hypercholesterolaemia, n (%)	79 (54)	51 (55)	14 (70)	7 (47)	7 (37)	0.19

**Characteristic**	**Overall**, N = 146	**DCB Only**, N = 92	**Stent**, N = 20	**BA**, N = 15	**Stent and DCB**, N = 19	**p-value**[1]
HTN, n (%)	83 (57)	50 (54)	15 (75)	9 (60)	9 (47)	0.30
PVD, n (%)	14 (9.6)	12 (13)	1 (5.0)	1 (6.7)	0 (0)	0.36
Stroke, n (%)	12 (8.2)	10 (11)	1 (5.0)	1 (6.7)	0 (0)	0.56
MI, n (%)	114 (78)	75 (82)	14 (70)	12 (80)	13 (68)	0.45
CABG, n (%)	8 (5.5)	4 (4.3)	4 (20)	0 (0)	0 (0)	0.05
Heart failure, n (%)	3 (2.1)	3 (3.3)	0 (0)	0 (0)	0 (0)	>0.99
AF, n (%)	17 (12)	11 (12)	2 (10)	1 (6.7)	3 (16)	0.92
FHx_CAD, n (%)	29 (20)	14 (15)	4 (20)	5 (33)	6 (32)	0.17
COPD, n (%)	10 (6.8)	7 (7.6)	1 (5.0)	0 (0)	2 (11)	0.78
Diabetes, n (%)	44 (30)	30 (33)	7 (35)	3 (20)	4 (21)	0.62
Smoking history, n (%)						0.48
Never Smoked	28 (19)	13 (14)	5 (21)	5 (23)	5 (18)	
Current/Ex Smoker	118 (81)	79 (88)	15 (79)	10 (77)	14 (82)	
GFR, Median (IQR)	83 (66 – 100)	87 (68 – 100)	66 (52 – 81)	77 (57 – 93)	95 (76 – 114)	**0.01**
Frailty score Median (IQR)	0.00 (0.00 – 0.70)	0.00 (0.00 – 0.63)	0.00 (0.00 – 1.45)	0.00 (0.00 – 0.20)	0.00 (0.00 – 0.65)	0.37

[1] Kruskal-Wallis rank sum test; Fisher's exact test; Pearson's Chi-squared test

Supplementary Table S4: Clinical and angiographic characteristics

Characteristic	**Overall**, N = 146	**DCB Only**, N = 92	**Stent**, N = 20	**BA**, N = 15	**Stent and DCB**, N = 19	**p-value**[1]
Presentation, n (%)						0.51
STEMI	129 (88)	82 (89)	16 (80)	13 (87)	18 (95)	
NSTEMI	17 (12)	10 (11)	4 (20)	2 (13)	1 (5.3)	
Timing of presentation, n (%)						**0.01**
Late	15 (10)	4 (4.3)	4 (20)	3 (20)	4 (21)	
Very Late	131 (90)	88 (95.7)	16 (80)	12 (80)	15 (79)	
Cardiogenic shock, n (%)	16 (11)	8 (8.7)	4 (20)	1 (6.7)	3 (16)	0.37
Intubation, n (%)	7 (4.8)	3 (3.3)	2 (10)	0 (0)	2 (11)	0.18
Cardiac Arrest, n (%)	19 (13)	13 (14)	3 (15)	2 (13)	1 (5.3)	0.82
Vessel Treated, n (%)						0.21
LMS	0 (0)	0 (0)	0 (0)	0 (0)	0 (0)	
LAD	67 (44.9)	40 (43.5)	8 (40)	10 (67)	9 (47)	
LCx	25 (17.1)	17 (18.5)	4 (20)	0 (0)	4 (22)	
RCA	49 (33.5)	33 (35.9)	5 (25)	5 (33)	6 (33)	
Graft	5 (3.5)	2 (2.1)	3 (15)	0 (0)	0 (0)	
True bifurcation, n (%)	64 (44)	39 (42)	8 (40)	6 (40)	11 (58)	0.61

Characteristic	Overall, N = 146	DCB Only, N = 92	Stent, N = 20	BA, N = 15	Stent and DCB, N = 19	p-value[1]
Aspirin, n (%)	143 (98)	90 (98)	20 (100)	15 (100)	18 (95)	0.59
P2Y12, n (%)						**0.002**
Clopidogrel	45 (31)	21 (23)	13 (65)	5 (33)	6 (32)	
Ticagrelor	95 (65)	69 (75)	7 (35)	9 (60)	10 (53)	
Prasugrel	3 (2.1)	1 (1.1)	0 (0)	0 (0)	2 (11)	
No	3 (2.1)	1 (1.1)	0 (0)	1 (6.7)	1 (5.3)	
Gp IIbIIIa, n (%)	108 (74)	68 (74)	16 (80)	9 (60)	15 (79)	0.55
Thromboaspiration, n (%)	84 (58)	50 (54)	12 (60)	9 (60)	13 (68)	0.71
Intravascular Imaging, n (%)						0.27
None	65 (44.5)	41 (44.5)	11 (55)	5 (33.3)	8 (42)	
OCT	42 (28.8)	31 (33.7)	2 (10)	5 (33.3)	4 (21)	
IVUS	39 (26.7)	20 (21.7)	7 (35)	5 (33.3)	7 (37)	
Previous Stent, n (%)						0.20
DES	74 (50.6)	47 (51.2)	9 (45)	10 (66.7)	8 (42)	
BMS	14 (9.6)	6 (6.6)	4 (20)	2 (13.3)	2 (11)	
DES and BMS	2 (1.4)	2 (2.2)	0 (0)	0 (0)	0 (0)	
BVS	1 (0.7)	0 (0)	0 (0)	1 (6.7)	0 (0)	

**Characteristic**	**Overall**, N = 146	**DCB Only**, N = 92	**Stent**, N = 20	**BA**, N = 15	**Stent and DCB**, N = 19	**p-value**[1]
unknown	55 (38)	37 (40)	7 (35)	2 (13.3)	9 (47)	
Vessel diameter, Median (IQR)	3.50 (3.00 – 4.00)	3.50 (3.00 – 4.00)	4.00 (3.44 – 4.00)	3.00 (2.63 – 3.50)	3.75 (3.25 – 4.00)	**<0.01**
Lesion length, Median (IQR)	26 (20 – 40)	26 (20 – 38)	25 (19 – 40)	20 (18 – 25)	40 (32 – 58)	**<0.01**
TIMI flow pre, n (%)						0.85
0	106 (72.6)	66 (72)	14 (70)	12 (80)	14 (74)	
1	7 (4.8)	4 (4.2)	1 (5.0)	1 (6.7)	1 (5.3)	
2	17 (11.6)	9 (9.8)	3 (15)	2 (13.3)	3 (16)	
3	16 (11)	13 (14)	2 (10)	0 (0)	1 (5.3)	
TIMI flow post, n (%)						0.21
0	0 (0)	0 (0)	0 (0)	0 (0)	0 (0)	
1	2 (1.4)	1 (1.1)	0 (0)	1 (6.7)	0 (0)	
2	13 (8.9)	12 (13)	0 (0)	0 (0)	1 (5.3)	
3	131 (89.7)	79 (85.9)	20 (100)	14 (92.7)	18 (95)	
Antiplatelet adherence, n (%)						0.69
No reported issues	137 (93.8)	86 (93)	19 (95)	15 (100)	17 (89)	
Issues Reported	9 (6.2)	6 (7)	1 (5.0)	0 (0)	2 (11)	

[1] Kruskal-Wallis rank sum test; Fisher's exact test; Pearson's Chi-squared test

Supplementary Table S5: Univariate Cox regression analysis for primary composite endpoint

**Cardiovascular mortality / ACS / TLR** (Univariate)	**HR (95% CI)**[1]	**p-value**
DCB-only vs DES (DES)	1.93 (0.96 to 3.89)	0.065
Male	0.71 (0.35 to 1.45)	0.34
Age	1.01 (0.98 to 1.04)	0.55
Hypercholesterolaemia	1.74 (0.89 to 3.37)	0.10
Hypertension	1.52 (0.79 to 2.92)	0.21
Peripheral vascular disease	1.73 (0.76 to 3.93)	0.19
Stroke	1.93 (0.81 to 4.62)	0.14
Myocardial infarction	1.58 (0.66 to 3.77)	0.30
CABG	2.08 (0.81 to 5.31)	0.13
Heart failure	0.00 (0.00 to Inf)	>0.99
Atrial fibrillation	0.52 (0.16 to 1.68)	0.27
Family history of IHD	0.88 (0.34 to 2.24)	0.78
COPD	1.50 (0.53 to 4.23)	0.44
Diabetes	1.95 (1.05 to 3.65)	**0.036**
Smoking history	0.90 (0.38 to 2.15)	0.81
GFR	1.00 (0.98 to 1.01)	0.70
Frailty Group		
Low	—	
Intermediate	2.16 (0.66 to 7.02)	0.20
High	0.00 (0.00 to Inf)	>0.99
Presentation		

**Cardiovascular mortality / ACS / TLR** (Univariate)	**HR (95% CI)**[1]	**p-value**
STEMI	—	
NSTEMI	0.91 (0.32 to 2.57)	0.86
Timing of stent thrombosis		
Late	—	
Very Late	0.25 (0.11 to 0.61)	**0.002**
Cardiogenic shock	1.14 (0.40 to 3.22)	0.81
Intubation	3.91 (1.19 to 12.9)	**0.025**
Cardiac arrest	1.15 (0.45 to 2.96)	0.77
Vessel treated		
LMS	—	
LAD	0.62 (0.18 to 2.12)	0.45
LCx	0.98 (0.27 to 3.52)	0.97
RCA	0.34 (0.09 to 1.30)	0.11
Graft		
True bifurcation	1.42 (0.76 to 2.64)	0.27
GP IIbIIIa	0.44 (0.23 to 0.83)	**0.012**
Thromboaspiration	0.78 (0.41 to 1.46)	0.43
Intravascular imaging		
OCT	0.63 (0.28 to 1.39)	0.25
IVUS	1.13 (0.55 to 2.35)	0.73
Previous Stent		
DES	—	

**Cardiovascular mortality / ACS / TLR** (Univariate)	**HR (95% CI)**[1]	**p-value**
BMS	1.71 (0.69 to 4.22)	0.25
BVS		
unknown	0.57 (0.28 to 1.16)	0.12
DES and BMS	0.00 (0.00 to Inf)	>0.99
Vessel diameter	0.77 (0.45 to 1.31)	0.33
Lesion length	1.01 (0.99 to 1.02)	0.47
Heavy calcification	0.67 (0.21 to 2.19)	0.51
Diffuse disease	1.08 (0.48 to 2.44)	0.86
Tortuosity	0.38 (0.09 to 1.58)	0.18
TIMI flow pre		
0	—	
1	1.79 (0.54 to 5.90)	0.34
2	1.16 (0.45 to 3.00)	0.76
3	0.84 (0.29 to 2.40)	0.74
TIMI flow post		
0	—	
1	0.00 (0.00 to Inf)	>0.99
2	0.63 (0.19 to 2.05)	0.44
3		
Issues with antiplatelet adherence	1.60 (0.57 to 4.53)	0.37

Supplementary Table S6: Clinical and angiographic characteristics

Characteristic	**Overall** N = 107	**DCB Only** N = 92	**POBA** N = 15	**p-value**[1]
Male	85 (79)	74 (80)	11 (73)	0.50
Age, Median (IQR)	68 (57 – 74)	66 (55 – 74)	71 (68 – 74)	0.059
Hypercholesterolaemia, n (%)	58 (54)	51 (55)	7 (47)	0.53
Hypertension, n (%)	59 (55)	50 (54)	9 (60)	0.68
Peripheral Vascular Disease, n (%)	13 (12)	12 (13)	1 (6.7)	0.69
Stroke, n (%)	11 (10)	10 (11)	1 (6.7)	>0.99
Myocardial infarction, n (%)	87 (81)	75 (82)	12 (80)	>0.99
CABG, n (%)	4 (3.7)	4 (4.3)	0 (0)	>0.99
Heart failure, n (%)	3 (2.8)	3 (3.3)	0 (0)	>0.99
Atrial fibrillation, n (%)	12 (11)	11 (12)	1 (6.7)	>0.99
Family history of CAD, n (%)	19 (18)	14 (15)	5 (33)	0.14
COPD, n (%)	7 (6.5)	7 (7.6)	0 (0)	0.59
Diabetes, n (%)	33 (31)	30 (33)	3 (20)	0.38
Current/Ex Smoker	89 (86)	79 (88)	10 (77)	0.38
GFR, Median (IQR)	84 (65 – 100)	87 (68 – 100)	77 (57 – 93)	0.38
Frailty Group, n (%)				>0.99
Low	101 (94.4)	86 (93.5)	15 (100)	
Intermediate	5 (4.7)	5 (5.4)	0 (0)	
High	1 (0.9)	1 (1.1)	0 (0)	
Presentation, n (%)				0.67
STEMI	95 (89)	82 (89)	13 (87)	
NSTEMI	12 (11)	10 (11)	2 (13)	
Timing of ST, n (%)				0.056
Late	7 (6.5)	4 (4.3)	3 (20)	
Very Late	100 (93.5)	88 (95.7)	12 (80)	
Cardiogenic Shock, n (%)	9 (8.4)	8 (8.7)	1 (6.7)	>0.99
Mechanical Ventilation, n (%)	3 (2.8)	3 (3.3)	0 (0)	>0.99
Cardiac Arrest, n (%)	15 (14)	13 (14)	2 (13)	>0.99
Vessel Treated, n (%)				0.19
LMS	0 (0)	0 (0)	0 (0)	
LAD	50 (47)	40 (43)	10 (67)	
LCx	17 (16)	17 (18)	0 (0)	
RCA	38 (36)	33 (36)	5 (33)	
Graft	2 (1.9)	2 (2.2)	0 (0)	
True Bifurcation, n (%)	45 (42)	39 (42)	6 (40)	0.86
Gp IIb/IIIa, n (%)	77 (72)	68 (74)	9 (60)	0.35
Thromboaspiration, n (%)	59 (55)	50 (54)	9 (60)	0.68
Intravascular Imaging, n (%)				0.56
None	46 (43)	41 (45)	5 (33)	
OCT	36 (34)	31 (34)	5 (33)	

**Characteristic**	**Overall** N = 107	**DCB Only** N = 92	**POBA** N = 15	**p-value**[1]
IVUS	25 (23)	20 (22)	5 (33)	
Previous Stent, n (%)				0.050
DES	57 (53)	47 (51)	10 (67)	
BMS	8 (7.5)	6 (6.5)	2 (13)	
BVS	1 (0.9)	0 (0)	1 (6.7)	
unknown	39 (36)	37 (40)	2 (13)	
DES and BMS	2 (1.9)	2 (2.2)	0 (0)	
Vessel diameter, Median (IQR)	3.50 (3.00 – 4.00)	3.50 (3.00 – 4.00)	3.00 (2.50 – 3.50)	0.052
Lesion length, Median (IQR)	26 (20 – 35)	26 (20 – 39)	20 (15 – 25)	**0.002**
Heavy Calcification, n (%)	12 (11)	10 (11)	2 (13)	0.67
Diffuse Disease, n (%)	19 (18)	16 (17)	3 (20)	0.73
Tortuosity, n (%)	14 (13)	11 (12)	3 (20)	0.41
TIMI flow PRE, n (%)				0.37
0	78 (73)	66 (72)	12 (80)	
1	5 (4.7)	4 (4.3)	1 (6.7)	
2	11 (10)	9 (9.8)	2 (13)	
3	13 (12)	13 (14)	0 (0)	
TIMI flow POST, n (%)				0.12
0	0 (0)	0 (0)	0 (0)	
1	2 (1.9)	1 (1.1)	1 (6.7)	
2	12 (11)	12 (13)	0 (0)	
3	93 (87)	79 (86)	14 (93)	
Aspirin, n (%)	105 (98)	90 (98)	15 (100)	>0.99
P2Y12, n (%)	26 (93)	21 (95)	5 (83)	0.39
Anticoagulation, n (%)	10 (9.3)	9 (9.8)	1 (6.7)	>0.99
Antiplatelet Adherence, n (%)				0.59
Nil Reported	101 (94.1)	86 (93.1)	15 (100)	
Issues Reported	6 (5.9)	6 (6.9)	0 (0)	

[1] Wilcoxon rank sum test; Fisher's exact test; Pearson's Chi-squared test
